# Multi-Channel Distributed Coordinated Function over Single Radio in Wireless Sensor Networks

**DOI:** 10.3390/s110100964

**Published:** 2011-01-17

**Authors:** Carlene E.-A. Campbell, Kok-Keong (Jonathan) Loo, Orhan Gemikonakli, Shafiullah Khan, Dhananjay Singh

**Affiliations:** 1 School of Engineering and Information Sciences, Middlesex University, London, NW4 4BT, UK; E-Mails: carlnjam@gmail.com (C.E.-A.C.); O.Gemikonakli@mdx.ac.uk (O.G.); skkust@hotmail.co.uk (S.K.); 2 Division of Fusion and Convergence of Mathematical Sciences, National Institute for Mathematical Sciences, Daejeon 305-390, Korea

**Keywords:** WSN, multi-channel, 802.11 DCF, MAC, channels

## Abstract

Multi-channel assignments are becoming the solution of choice to improve performance in single radio for wireless networks. Multi-channel allows wireless networks to assign different channels to different nodes in real-time transmission. In this paper, we propose a new approach, Multi-channel Distributed Coordinated Function (MC-DCF) which takes advantage of multi-channel assignment. The backoff algorithm of the IEEE 802.11 distributed coordination function (DCF) was modified to invoke channel switching, based on threshold criteria in order to improve the overall throughput for wireless sensor networks (WSNs) over 802.11 networks. We presented simulation experiments in order to investigate the characteristics of multi-channel communication in wireless sensor networks using an NS2 platform. Nodes only use a single radio and perform channel switching only after specified threshold is reached. Single radio can only work on one channel at any given time. All nodes initiate constant bit rate streams towards the receiving nodes. In this work, we studied the impact of non-overlapping channels in the 2.4 frequency band on: constant bit rate (CBR) streams, node density, source nodes sending data directly to sink and signal strength by varying distances between the sensor nodes and operating frequencies of the radios with different data rates. We showed that multi-channel enhancement using our proposed algorithm provides significant improvement in terms of throughput, packet delivery ratio and delay. This technique can be considered for WSNs future use in 802.11 networks especially when the IEEE 802.11n becomes popular thereby may prevent the 802.15.4 network from operating effectively in the 2.4 GHz frequency band.

## 1. Introduction

Wireless Sensor Networks (WSNs) [1–4] are used over a wide range and in varying fields such as military application, environmental monitoring, medical care, smart buildings and other industries. WSNs sensors are generally deployed randomly in the field of interest, delivering myriad types of events from simple periodic reports to unpredictable bursts of messages triggered by external events that are being sensed. These sensor nodes will work collaboratively to sense a given environment, perform in-network computations and communicate with a base station when a targeted event occurs. A large number of WSN based applications are emerging when compared with conventional wireless networks. WSNs also have several defined characteristics including limited transmission bandwidth, limited computation capability of individual nodes and limited energy supply. The current WSNs paradigm also has some interesting features including self-organization, dynamic network topology and multi-hop routing. These are important features for many real world applications nowadays.

The 802.15.4 standard defines a protocol for Low Rate Wireless Personal Area Networks (LR-WPAN). This allows for low cost of components, reduced coverage area, low transmission power, low bit rate and energy consumption [5]. The 802.15.4 PHY layer can operate at 868 MHz, 915 MHz and 2.4 GHz bands. The network bandwidth is very limited and the MAC layer packet is very small with a typical size of 30–50 bytes compared to 512 bytes in the 802.11 networks. 802.15.4 networks typically operate at 2.4 GHz Industrial, Scientific and Medical (ISM) band, which is used by popular 802.11 networks as well.

A number of researchers [6–13] have used a combination of both the 802.15.4 and the 802.11 networks within WSNs for comparison and evaluation purposes considering different scenarios, or 802.11 is used as an access point (AP) and at cluster heads to relay 802.15.4 sensor network data to sinks and other network servers and applications. When 802.15.4 and 802.11 are using the same channels, their CSMA/CA functions enable them to share time slots. However, using the same channels will cause 802.15.4 to suffer long delays while having 802.11 with a higher frequency range provides priority access of the channel in most cases. An overlap between them may adversely impact on the operation of 802.15.4, since it is a low power protocol which uses a small channel width compared to the transmitted power levels and channel width used by 802.11. The frequency band in which such interference issues are nowadays more critical for wireless networks.

The 802.11 standard [14] defines a communication protocol for wireless local area networks (WLANs), providing a total of 14 frequency channels, each of which is characterized by 22 MHz bandwidth. The fundamental media access method of the 802.11 is a DCF known as Carrier Sense Multiple Access with collision avoidance (CSMA/CA). It is a contention-based protocol that concentrates on the collisions of transmitted data and was developed mainly for wireless networks. Applying a multi-channel assignment to this scheme would help to reduce contention for a single medium, collision and congestion.

Multi-channel as it relates to wireless networks is used to assign different nodes to different channels in real-time transmission. This gives rise to having communications on different frequency bands. When sensor nodes are densely deployed, single channel MAC protocols may be inadequate due to a higher demand for the limited bandwidth. There have been a number of proposed MAC protocols lately, in order to improve network performance in WSNs using multi-channel assignments [15–28].

Our research is focusing on the design of multi-channel communication based on the 802.11 DCF over a single radio for WSNs in order to improve its communication performance namely throughput, end-to-end delay and channel access delay. Multi-channel protocols utilize bandwidth better and thus may perform favorably in cases of applications demanding high data rates. The 802.11 standards provide up to 12 non-overlapped channels, respectively, in 2.4 GHz and 5 GHz spectrums. Nodes within the transmission range of each other can operate on different non-overlapped channels so as to avoid interference. We considered the following factors in focusing on using 802.11 for WSNs:

- Like 802.15.4, the 802.11 DCF operations are also based on the CSMA/CA algorithm. It can be used for a wireless sensor surveillance system that is low-cost, reliable, easy to manage, easy to deploy and can process video data for automated real-time alerts. Despite much attention in recent years, researchers have yet to achieve the goal of long term, independent operations of sensor network deployments under this constraint.
- 802.15.4 is applied to low data rate and short distance communication sensor networks where topology of a sensor network changes very frequently. Having 802.15.4 and 802.11 operating within the same frequency band may become problematic when 802.11n networks are in used. 802.11n has several new features such as the use of multiple input and output streams (MIMO) and channel bonding that would allow the data rates up to 450 Mbps to be achieved. In particular, channel bonding refers to the use of a 20 MHz wide extension channel in addition to the control channel used by 802.11 networks. At high traffic loads, an 802.11n network would use a total bandwidth of 40 MHz when operating in 2.4 GHz band. Two or more 802.11n networks operating in the same location with an 802.15.4 network would leave no 802.15.4 channel free from 802.11n interference.

The rest of this paper presents related work, the proposed system model and how nodes are assigned to channels, simulation results and the performance analysis. Finally the conclusion and future work is presented.

## 2. Related Work

Multi-channel assignment for WSNs has been studied by a number of researchers. The hybrid approach studied in [15] are similar wherein each node has a fixed interface on a common channel which is used for package control and exchange while the other interfaces are switched among the remaining channels for data transmission.

Other hybrid multi-channel protocols in [16] consist of two parts wherein one part handles MAC issues such as queuing, switching and broadcast and the second part is a distributed assignment algorithm. These models maintain a table which records the channels being used by its neighbors. In this technique, nodes constantly check the table in order to determine the number of nodes assigned to a channel. In [15], they also proposed a hybrid approach for each semi-fixed channel assignment, a heuristic algorithm used based on transferring from a coloring based problem.

In [16–19], static and dynamic strategies were used to assign channels. In [15], a load-aware channel assignment was proposed. In [15,20–23], multi-channel MAC protocols were proposed; these protocols either require multiple radio transceivers at each node or certain kind of messages for channel negotiation. However, using multiple transceivers require the use of energy which is a constraint in WSNs. In this case channel negotiation packets are not seen as a small overhead. Both TMMAC [24] and MNSN [25] are multi-channel MAC protocols designed for WSNs. They are protocols that were designed to assign different channels to nodes in a two hop neighborhood so as to avoid potential interferences. Simulation results show that they improve performance compared to single channel protocols. The downside is that a node has a different channel from its downstream and upstream nodes. In the multi-hop flow, nodes have to switch channels in order to receive and forward packets. This causes frequent channel switching and potential packet losses. In order to prevent packet loss these protocols use some negotiation or scheduling schemes to coordinate channel switching and transmission among nodes with different channels. The challenges they face are that they need many orthogonal channels for channel assignment in dense networks; they also require precise time synchronization at nodes with frequent channel switching delays and scheduling overheads especially for high data traffic. In [23], empirical experiments with Micaz motes were done to show that node-based protocols may not be suitable for WSNs in practice.

In [26], a channel scheduling mechanism is used to manage and decide when a node should switch channel to support the current communication requirements. They also adopt the graph base approach.

Ozlem *et al.* [27] proposed a multi-channel scheme based on LMAC which allows the node to utilize new frequency channels on-demand, if the network reaches a density limit. This method is composed of two phases, one where the nodes try to select timeslots according to the single channel in LMAC rule and the second involves nodes which are unable to grab a timeslot in the first phase invite the neighbor nodes which are free to listen to them on an agreed channel or time slot.

Nasipuri’s scheme [28] was one of the first multi-channel CSMA protocols that used channel reservation. If there are *N* channels, the protocol assumes that each node can monitor all *N* channels simultaneously with *N* transceivers. This multi-channel scheme was just a simple extension from the single channel 802.11 MAC, which requires each node to have *N* transceivers with one for each channel; this was not feasible for a practical system.

## 3. Proposal for MC-DCF

This approach will use multi-channel assignments in 802.11 DCF over a single radio for WSNs known as MC-DCF. Node interface will be able to switch between channels. The approach will have all nodes aware of the channels in use but each node interface can only tune into one channel at a given time. At initialization, a random assigner that employs uniform distribution will be applied to distribute node interfaces to channels. This ensures that each channel will have about the same number of neighboring nodes assigned to it at start up. A number of approaches that have been used are highlighted in the related work. This will increase the number of nodes that are granted access to the wireless medium. In this approach nodes will switch channel when the contention window of DCF has reach an assign threshold. The sink node will perform interface switching in order to receive data from channels coming from source nodes. If there is a collision, the MAC method will invoke the backoff procedure implemented within the MAC protocol. Nodes will only monitor activities on the current channel they are assigned to and when switched to another channel it will listen to the signal within range and update itself. If all the channels are busy they will revert to backoff state.

### 3.1. Existing Challenges

There were existing challenges in WSN that needed to be considered when operating in the 802.11 network:

- Interferences among neighboring nodes
- Bandwidth limitation
- Contention of the medium
- Hidden terminal node
- 802.11 DCF decreases considerable in multi-hop network due to collision
- Single channel architecture
- Limited in power, computational capacities and memory
- Topology of sensor network changes very frequently
- Sensor nodes mainly use a broadcast communication paradigm instead of point-to-point communications
- Sensor nodes are densely deployed and are prone to failures
- Number of sensor nodes can be several orders of magnitude higher than nodes ad hoc networks
- Non-overlapping channels used by 802.15.4 and 802.11 in the 2.4 frequency band
- WSN typically deployed for periodic monitoring process not for streaming data
- Design for low data rate to save energy as replacement batteries are very cumbersome to replace or may not be able to be replaced.

These issues are considered in our simulation set-up.

### 3.2. IEEE 802.11 DCF Backoff Procedure

**Figure 1.**
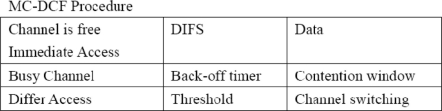
MC-DCF procedure.

The original random backoff timer is invoked when finding a medium busy by the carrier sense (CS) mechanism of the DCF. This will be modified to invoke channel switching based on a set threshold criteria. The implementation will be done in NS2 for multi-channel, single-radio by using the existing MACs protocol stack and the work done by for cognitive radio cognitive network (CRCN) GUI, SNR lab/Michigan Technological University and the Hyacinth model. In the designs, multiple channel objects have been created through the TCL library. Nodes will be switching to different channel objects during the simulation process. During network initialization all nodes will be made known of the channels by a channel notifier. The channel sensor invokes the random assigner after the channel notifier updates node of all channels on the network and will uniformly distribute radios of nodes to a channel in a load balancing format. When a node intends to transmit and senses that the medium busy, it will back-off and re-try. If the contention window threshold is reached the sensor will invoke and channel switching begins. Nodes will switch to other channels in order to check if they are busy. If another channel is free, a node will update itself off its neighboring node on the same channel and will transmit based on the MC-DCF procedure in Figure 1.

### 3.3. Multi-Channel

In our approach, we have utilized the dynamic assignment. As regards the dynamic assignment, each node is assigned a channel for data transmission, once sending data the node will not be able to switch channel. It will be able to switch channel if searching for an available channel to transmit and update its information about neighboring nodes on the same channel. Each node will know the number of channel available for switching at initialization. Having a dynamic assignment utilizing a single interface can provide significant performance benefits over a static approach as it can potentially utilize instantaneous traffic or interference information and reduce wastage of the precious already limited bandwidth. Due to the fact that WSN cannot provide reliable and timely communication with high data rate requirements over single channel because of interference, radio collision and limited bandwidth, since they are mainly for nodes placed in a remote area that periodically send data to the host. Why multi-channel over 802.11 DCF which uses high data rate? As mention before WSN is an emerging technology that has become one of the fastest growing areas in the communication industry. The demand for using this medium is increasing with a wide range of deployment for monitoring, surveillance systems and other multimedia systems such as streaming or real time data. With this in mind, we utilized the 802.11 standard which uses a range of data rate. When the 802.11n becomes popular, two or more 802.11n networks operating in the same location with an 802.15.4 network would leave no 802.15.4 channel free from 802.11n interference.

### 3.4. Channel Switching

Nodes are not bound to a particular channel and have the option of switching between channels. Channel switching among sending nodes will only occur after a set threshold has been reached during the backoff period. The radio will not switch during transmission, this can cause data packet to get lost or corrupted. Therefore, during transmission the radio will remain on the channel until completion of transmission. However, at the receiving/sink node channel switching will be more intense as the sink node will be receiving data from more than one source nodes. The switching delay incurred will depend on the packet size being received at each interface. Consider for example a data packet of 1kb being accepted:

- The time taken by 1 second would be 54 × 10^6^
- Time required to transmit one bit = packet size/data rate
- Time taken by 1 bit $\frac{8,000\;b}{54\times 10^{6}\mathrm{bps}}$
- The radio will take 160 μs to switch to the next channel

We will study the impact of channel switching from simulation scenarios between the sources that are sending data directly to the sink.

### 3.5. System Modeling

In order to develop sensor algorithms for assigning all channels to node interfaces, channel checking and switching, this proposal has utilized the 802.11 DCF contention based protocol where decisions are made base on the window size and backoff algorithm on multiple non-overlapping channels over single radio. The problem in a contention based network is that all nodes contend for the same medium. Multi-channel will have nodes contending for greater than one channel instead of a single channel. The backoff mechanism of DCF cannot provide deterministic upper bound channel access delay for sensor networks. The contention and backoff strategy is unfair to the already existing nodes that are backing off. Implementing channel sensor and switching within the backoff mechanism will eliminate the unfair strategy on the backing off for all nodes and a node will only keep updated with its neighboring nodes with range on the same channel. This approach is a novel one that has not been done by any other research to the knowledge of this author. The overall goal for this design is to have multi-channel sensor network with 802.11 so that nodes can switch channels and prevent severe delay, packet losses, increased throughput and having nodes options of channel to transmit, with no central scheduler to assign channels. As constant traffic sources cannot be assumed at all times and traffic can be bursty in nature.

### 3.6. Design Approach

NS-2 is used as the simulation platform. At initialization, all nodes are made aware of the number of channels available through a channel notifier. When a node requires transferring data, the Carrier Sense (CS) mechanism is invoked in order to determine if the channel is busy or idled. If the CS is zero, this indicates that the channel is idled; otherwise the channel is busy and will be determined as transmitting data. During the backoff period of the original DCF, the contention window (CW) parameter will take an initial value of the control window (CWmin). The CW will take the next value in series every time an unsuccessful attempt to transmit causes the retry counter to increment. When the CW reaches maximum (CWmax), it will remain until the window is reset. In the proposal model the retry counter will reach its threshold after the third attempt and switch channel based on the design parameters. Nodes will enter wait state if all channels are busy.

**Figure 2.**
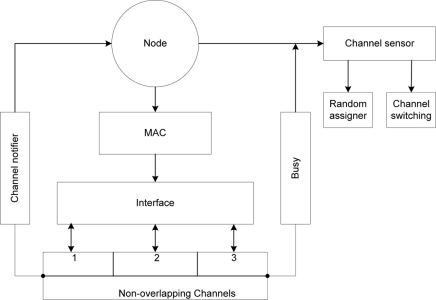
MC-DCF design model.

In Figure 2 during initialization of the network, the channel notifier uses combined functions from the management system in order to obtain information regarding the number of interfaces at the upper layer by invoking a logical communication with the distribution system medium (DSM) [14] at the MAC sub layer. The channel notifier will assume that all nodes are in the same basic service set (BSS) and broadcast all the available channels within the BSS. The random assigner and channel switching is under control of the channel sensor that references the channel notifier. The sensor invokes the random assigner after the channel notifier updates nodes of all channels on the network. The assigner keeps a count of interfaces from which a uniform distribution algorithm mandates the proportionality for each channel *i.e.*, how many interfaces to a channel. The random assigner randomly assigns interfaces to channels during the initialization process. Each node keeps updated information of its neighbors on the same channel within range by sensing the medium periodically and learning about the medium through the virtual carrier sense mechanism [14]. The CS also determines the busy/idle state of the medium.

During channel assignment where *C* is the number of non-overlapping channels available and *N_i_* is the number of nodes interfaces to be assigned to channels within the IBSS (B). *C* = (*C_1_*, *C_2_*, *C_3_*,*…C_n_*), where *n* is the channel number *N* = (1, 2, 3,...*i*) where *i* is the total number of node interfaces. The calculated uniform distribution equation is:

(1) $$\frac{\Sigma_{i=1}^{C}N_{i}}{C}$$

**Figure 3.**
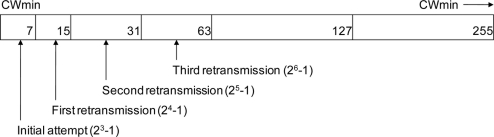
Contention window with defined threshold 26-1.

When a station (STA) wishing to transmit and sense busy, the CW shall take the next value in series every time an unsuccessful attempt to transmit causes the STA retry counter to increment. The channel sensor shall maintain a retry counter and after the define threshold is reached; it will invoke the channel switching parameter. Figure 3 shows the contention window with the define threshold 26-1.

**Figure 4.**
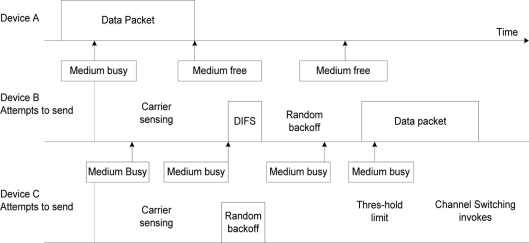
Contention period and channel switching.

In Figure 4, if node A is transmitting a data packet and node B senses that the medium is busy and waits, after the initial attempt of waiting to transmit it senses the medium on the channel being used, attempts on the first retry and detects that the medium is free of other transmissions. The node waits for a predetermined DIFS (distributed inter-frame spacing) period, once it senses no other transmission before the end of the DIFS period, it computes a random backoff time between values of CWmin and CWmax then commences its transmission. Node C senses the medium to be busy and makes repeated attempts to transmit a packet; the computed backoff period is doubled with each attempt until the specified threshold is reached. When the threshold is reached the channel sensor invokes the channel switching. The node interface will tune to another channel, senses if the medium is busy. If busy, it will switch otherwise it will proceed with transmission. If all channels are busy the node will revert to random backoff time and set its backoff timer using the equation in [14].

In Figure 2, the MC-DCF model is proposed for WSN. This multi-channel model is a contention based technique in a carrier sensed co-coordinated function process. This multi-channel backoff model brings added qualities of the diverse MAC resolution mechanisms of WSN. It is made up of three different MC-DCF techniques: channel notifier, channel sensor and the non-overlapping channels. These techniques allow nodes to be aware of the available channels, switch to another channel and to enter wait state when no channel is available.

**Figure 5.**
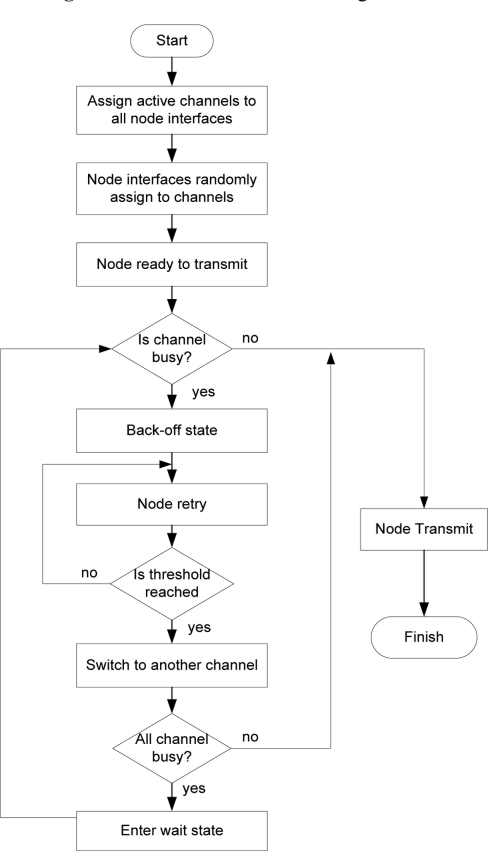
Flow chart for channel assignments.

Contention based techniques are best resolved by preventive methods but are most difficult to predict due to all nodes contending for a single channel. These clearly indicate that multi-channel with switching control systems as shown in Figure 5 will provide the needed best overall practice access control in accessing the medium while reducing collision, delay and the hidden node problem in the WSN. The idea is to achieve a multi-access, simultaneous transmission and maintain a good quality of communication which can be obtained as long as the distance between the sensor node and the sink node are short enough and the adequate strength of the signals are received.

The challenges in the MAC channel access control, highlighted previously in this paper, and the DCF backoff algorithm coupled with the channels within the 2.4 frequency band can be mitigated by the combination and integration of the MC-DCF Models as shown in Figure 2 and Figure 5 and further linking them to other application in 802.11 WLAN and ad hoc network systems.

## 4. Results and Discussion

### 4.1. Simulation Procedure

MC-DCF being the propose protocol for Multi-channel Distributed Coordinated Function, will be simulated using the NS-2 simulation platform. As mentioned before, at initialization all nodes will be made aware of the number of channels available. When a node wants to transfer data the Carrier Sense (CS) mechanism is invoked in order to determine if the channel is busy or idled. During the backoff period the contention window (CW) parameter will reach its threshold after the third attempt and switch channel. A node will enter a wait state if all channels are busy.

Our aim is to investigate multi-channel performance within a single-hop (the link quality), *i.e.*, the packet reception rate. In this paper, we analyzed the performance of the MC-DCF protocol by extensive simulations with NS2. Different simulation scenarios are studied according to three different performance metrics: aggregate throughput, delivery ratio and access delay.

The sensor nodes are randomly placed in a 1,000 × 1,000 m^2^ area. The radio range is set to 50 m. The radio bandwidth is 2 Mbps. The number of nodes is 100. The number of channels ranges from 3 to 10, since the spectral mask only defines power output restrictions up to ±11 MHz from the centre frequency to be attenuated by 30 dB. It is often assumed that the energy of the channel extends no further than these limits.

The 802.11 channels are effectively 22 MHz wide, the consequence is that stations can only use every fourth or fifth channel without overlap, typically 1, 6 and 11 in the Americas, and in theory, 1, 5, 9 and 13 in Europe although 1, 6, and 11 are typical there too. However if transmitters are closer together, overlap between the channels may cause unacceptable degradation of signal quality and throughput. The MAC protocols are 802.11 DCF and MC-DCF.

The simulation time for each scenario is 500 s. The aggregate throughput is calculated as the total amount of data delivered to the sink per unit time by the MAC protocol and is computed as:

(2) $$\text{Aggregate throughput}=\sum_{i=1}^{n}\left(R_{i}\times \frac{B}{t}\right)$$

where n is the number of receiver R, throughput is B/t, B is the bytes received by a receiver i in some duration of time and i = {1, 2, 3,...,n}. The delivery ratio is the ratio of total number of packets received by the nodes to the total number of packets transmitted times the number of receivers and is computed as:

(3) $$\frac{\Sigma_{i=1}^{n}R_{i}}{\Sigma_{i=1}^{n}s_{i}}$$

where *S_i_* means total data size of CBR packet node i sent, *R_i_* means total data size of CBR packet node i received.

The access delay is the backoff time used in DCF [14], the access delay can also be calculated as the packet size × 8 (1 byte) divided by the link size plus the propagation delay that is

(4) $$\frac{\text{packet size}\times 8}{\text{link size}}+\text{Propagation delay}$$

Nodes only transmit to neighboring nodes within range, transmitting over a wider range may consume more energy which is not desired by WSN and also to eliminate communication interference and hidden node problems [2].

### 4.2. Performance Analysis of the Proposed MC-DCF

In our simulation scenarios we do not assume large networks that are densely deployed; we consider a network for sensor surveillance system with continuous streaming data. Surveillance systems are mainly deployed for organization, parks and vehicular traffic not for remote monitoring. In this instance nodes will always be static and powered and as such the depletion of battery life is not considered. We will be simulating CBR traffic to be sent every 2 second to prevent buffer overflow and to replicate streaming data. The default data rate for MC-DCF will be 2 mbps.

**Figure 6.**
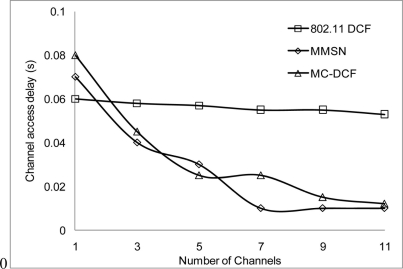
Delay impact on protocols.

**Figure 7.**
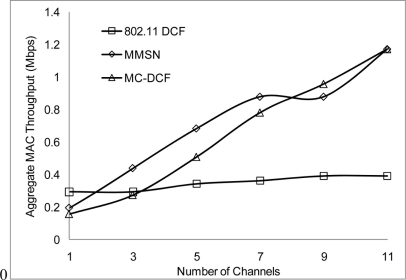
Throughput impact on protocols.

**Figure 8.**
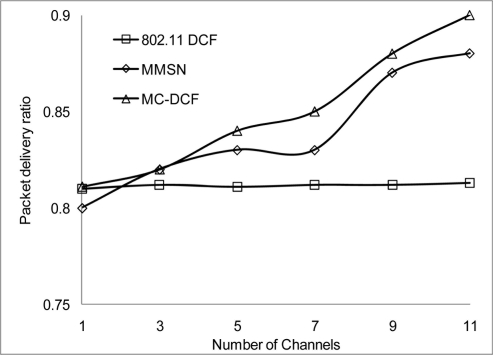
Delivery ratio impact on protocols.

In Figures 6, 7, and 8, we analyzed the performance of the number of channels on the 3 mentioned metric against MMSN [25]. Zhou *et al.* introduced the MMSN multi-frequency MAC protocol that was designed for WSN. It is a slotted CSMA protocol and at the beginning of each timeslot nodes need to contend for the medium before they transmit. We observed that channel access delay and aggregate throughput show MMSN perform slightly better than MC-DCF. MMSN uses a small packet size of 30–50 bytes, which contribute to the performance observed. This gives evidence that MC-DCF will outperform MMSN in the 802.11 network, should both protocols operate within the data rate (from 2 Mbps up to 54 Mbps) of the 802.11 networks. 802.11 DCF does not show much variation as this protocol only operates on a single channel. In Figure 8, MC-DCF showed a better performance in packet deliver ratio to that of MMSN. This is due to the fact that the advantage of higher data rate has more packets delivering and receiving by each node.

**Figure 9.**
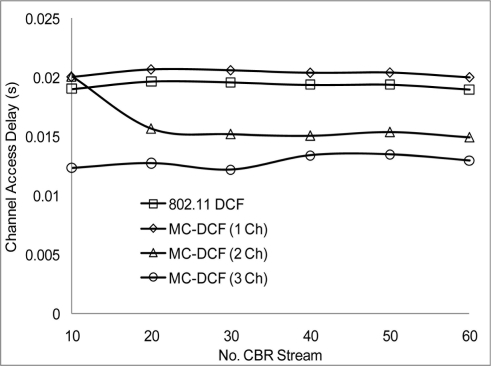
Delay impact on CBR streams.

We now studied the impact of the number of non-overlapping channel in the 2.4 frequency band of the 802.11 network. We varied the number of CBR streams, node generate packets toward the sink node, with the option to switch channel base on the MC-DCF backoff algorithm criteria. In Figure 9 when channel load reaches about 30 CBR stream, there is not much effect as it relates to delay in 802.11 DCF and when MC-DCF uses 1 channel. Streaming too many data flow, using a single channel shows that the system becomes saturated from backing off and buffer overflow. MC-DCF and 802.11 DCF shows similar delay pattern. However having more than 1 channel show a reduction in delay in accessing the channel and with the channels coping with more load and after 45 CBR before becoming saturated.

**Figure 10.**
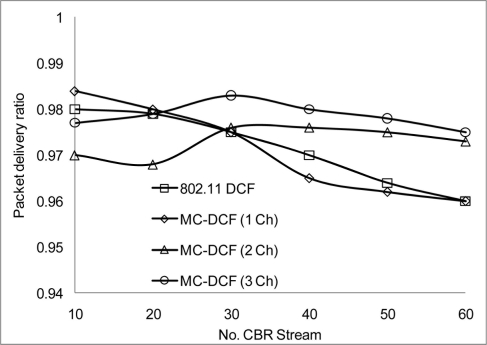
Delivery ratio impact on CBR stream.

**Figure 11.**
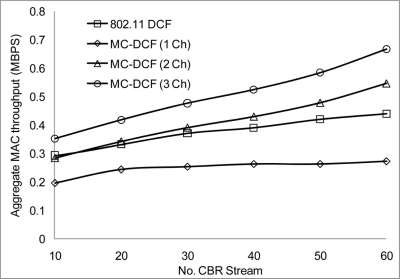
Throughput impact on CBR streams.

Figure 10 shows that the delivery ratio when 3 channels are used have more packets delivered compared to 1 and 2 channels. Having 1 channel as can be seen in 802.11 DCF and MC-DCF (1 Ch), experience constant degradation as the CBR streams increases. This degradation results in constant backing off where nodes are contending for the same channel with results in more packet loss. In Figure 11, a similar trend is seen where MC-DCF with 3 channels has a better aggregate throughput, where more data are deliver to the receiving node. MC-DCF with single channel performs worse with more unsuccessful attempts having less data delivered to the receiving node. This showed that with the modification to the backoff algorithm where nodes have an option to switch channel, if this procedure remained while using a single channel, backing off becomes more frequent as the threshold is reached much quicker. In using a single channel the original 802.11 DCF performed better than the MC-DCF single channel, the original DCF had the option of reaching the maximum CW window size before entering wait state.

**Figure 12.**
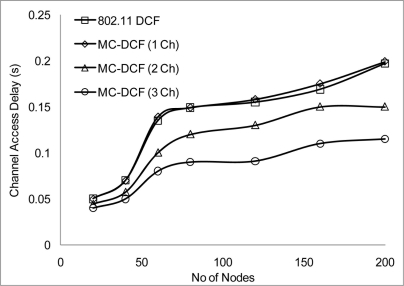
Delay impact on node density.

We have conducted more simulation where we varied the number of nodes sending CBR streams every 2 seconds in order to analyze the density effect of the network. MC-DCF performed better when nodes have 3 channels to transmit on simultaneously. Figure 12 shows 802.11 DCF and MC-DCF experiencing higher delay as more nodes transmit more packets and the network become denser. When 2 or more channels are transmitting as can be seen in Figure 12 there is a relative improvement in delay.

**Figure 13.**
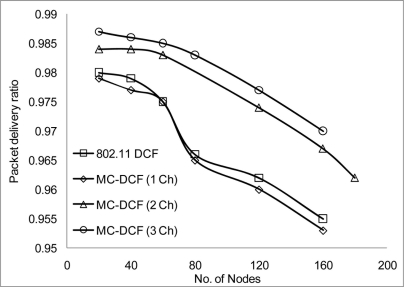
Delivery ratio impact on node density.

**Figure 14.**
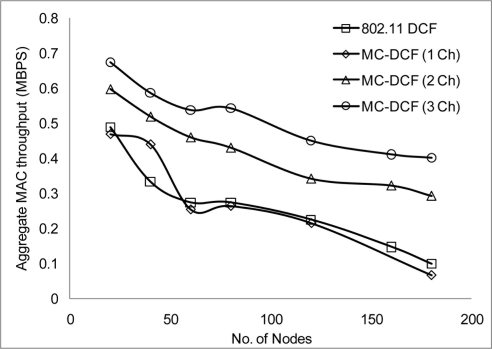
Throughput impact on node density.

The packet delivery ratio and the aggregate throughput in Figure 13 and Figure 14 respectively show a better performance when 2 or more channels are used. Although there is a better performance using 2 or more channels, the more nodes transmitting packets through the network, the performance starts degrading. This is not unusual as nodes will be switching channels, backing off and entering wait state which is the norm in a contention base network. However, from Figure 13, the system performance is still within limit to transmit streaming data for WSN within the 802.11 network. An average of approximately 2.1% degradation of packet delivery ratio is experienced with a total of 97% delivery ratio for 3 channels. The aggregate throughput as a function of the offered load for 3 channels showed a throughput decrease of 14%.

**Figure 15.**
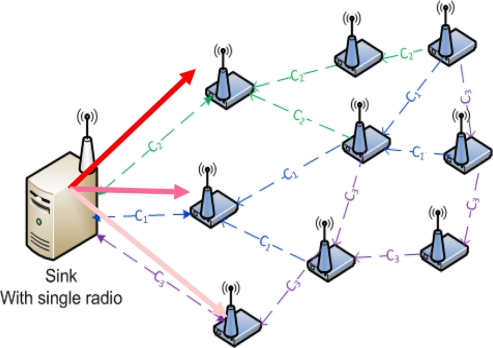
Sink node with single radio doing channel switching.

Figure 15 shows a sink node with a single radio switching between channels in order to receive data from more than one source nodes. We observed channel switching performance at the sink by varying the number of source nodes the sink needs to receive data. Here, we considered the access delay and packet delivery ratio.

**Figure 16.**
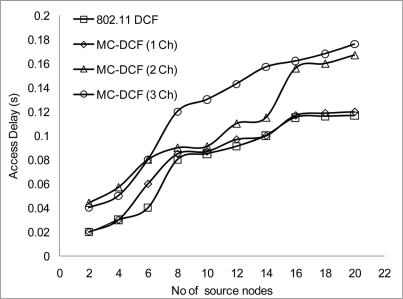
Delay at sink receiving data from source nodes.

**Figure 17.**
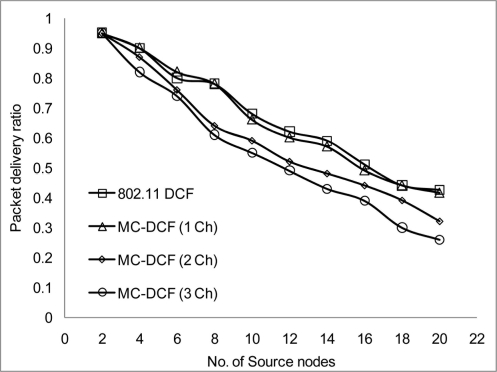
Delivery ratio at sink receiving packets from source nodes.

**Figure 18.**
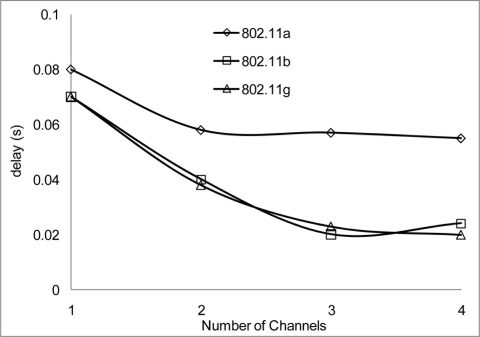
Delay occur at 50 m range and data rate of 2 Mbps.

**Figure 19.**
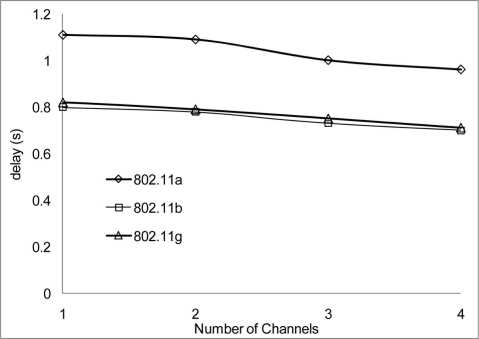
Delay occur at 100 m range and data rate of 2 Mbps.

In Figures 16 and 17, we examined the effect of the sink receiving data directly from sources within its range that are sending data to be accepted. From our observation, the more sources delivering to the sink the more delays are encountered and the packet delivery ratio decreases in a similar manner. This is due to the sink node having to be constantly switching between channels in order to receive data, which incur severe switching delay plus the time taken to accept data before switching. The aggregation throughput degradation that has been observed in previous simulation within 2 or more channels can be accounted for mainly at the sink node where severe delay has been encounter. This results in drop packets. More work will be done in this area in order to improve delivery of packets from the source to the sink in a multi-channel environment. Figures 18 and 19 show that 802.11 DCF and MC-DCF with a single channel gave a better performance than the multi-channel protocols. This is due to the fact that the sink node is operating in a single channel mode with no extra overhead and switching delay occurring when receiving data.

### 4.3. Performance Analysis of 802.11a/b/g

With the above simulations, we have seen that WSN can operate in 802.11 networks for sensor surveillance system with continuous streaming data which is not densely deployed. Nodes will always be static and powered and as such the depletion of battery life is not considered. Consider that 802.11n network may cause severe problems within 802.15.4 and 802.11 operating within the same frequency band because the 802.11n has several new features such as the use of multiple input and output streams (MIMO) and channel bonding that would allow the data rates up to 450 Mbps to be achieved. We conduct simulations to analyze the signal strength with different data rate over the 802.11a/b/g networks. We will also look at metrics such as delay, delivery ratio and aggregate throughput. The performance among the networks will aid in determining the range, data rate and preferable 802.11 networks to operate WSN not necessarily streaming surveillance system for future problems that may arise with 802.11n.

In our simulation scenarios, we will consider sensor nodes placed in a 1,000 × 1,000 m^2^ areas, the radio range and radio bandwidth with each scenario in order to determine suitable signal strength when operating in the 802.11 network for WSN. The number of nodes is 100 and the number of non-overlapping channels is 4 using the UK 2.400–2.4835 GHz frequency band. We configured the proposed MC-DCF MAC protocol to operate with the 802.11 a/b/g network for channel assignment and switching the multichannel. We will be analyzing the impact on 802.11 a/b/g and the radio range.

**Figure 20.**
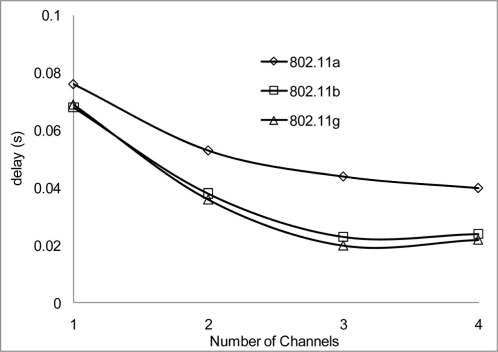
Delay occur at 50 m range and data rate of 10 Mbps.

The experiment results in Figures 18 and 19 show the delay that occurs when simulating 2 Mbps over a 50 and 100 m range for 802.11 a/b/g. Figure 20 shows that 802.11 a experienced a high level of delay compared to 802.11 b/g which shows slight variation in delay among them. Similarly at 100 m range the delay among the networks 802.11 a/b/g show an increase in delay among them all with 802.11 a experiencing high delay. The high delay experience by 802.11 a is as a result of it not being backward compatible to 802.11 b and it was design to operate at a minimum data rate of 6 Mbps. So operating with a data rate of 2 Mbps causes possible frequent dropped connections and degradation of service. The increase in delay that is experienced by all networks in Figure 19 indicate that at 100 m range among nodes experiences weak signal which makes it difficult for transmission and as such degradation of the networks.

**Figure 21.**
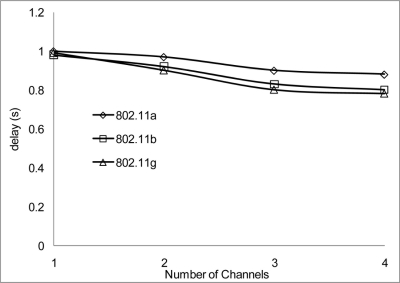
Delay occur at 100 m range and data rate of 10 Mbps.

Figures 20 and 21 show the delay that occurs when simulating at 10 Mbps. A similar pattern in performance is experienced at the 100 m range where the degradation of the networks are much higher. However, 802.11 a also shows an improvement in delay, this indicates that 802.11 a operates better at 6 Mbps and above but 802.11 b/g gives a better performance which shows that if signal quality becomes an issue 802.11 b/g can scale back to lower transfer data rate.

**Figure 22.**
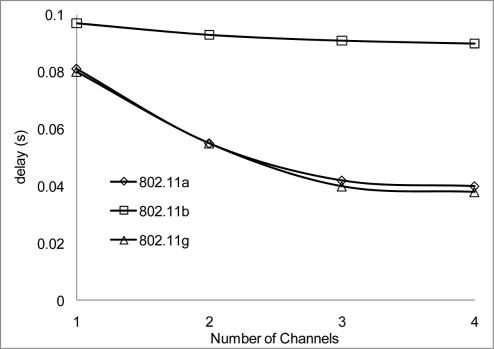
Delay occur at 50 m range and data rate of 54 Mbps.

**Figure 23.**
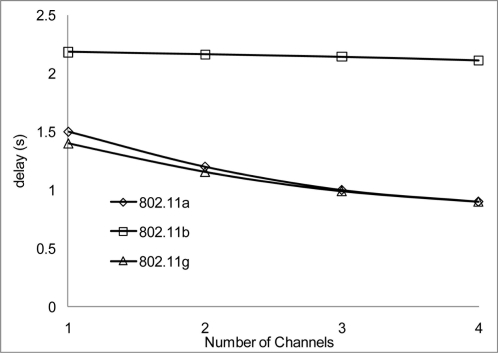
Delay occur at 100 m range and data rate of 54 Mbps.

Figures 22 and 23 show the delay that occurs at 54 Mbps. Both 802.11 a/g show a better performance than 802.11 b, which seems not to show any improvement during the simulation over all the channels. This clearly showed that 802.11 b cannot operate with data rate higher than 11 Mbps. Also from the simulation results the data rate does not make a positive impact regarding operating at 100 m range. At 100 m range the networks experience high delay which degrades the system significantly.

Throughout the group of simulations, the impact on delay over different range and channels show that a better performance is achieve at the 50m range to that at the 100 m range in delays. Also when 2 or more non-overlap channels within the 2.4 frequency band are used, there are even better performances achieve, which are evident in Figures 18, 20 and 22.

The performance results on the aggregate throughput are shown below. The results have been simulated over the 50 and the 100 m range with different data rates, 2, 10 and 54 Mbps for 802.11 a/b/g on 4 non-overlapping channels. The results show a similar pattern where the 50 m range gives a better performance having more data deliver at the receiving nodes.

**Figure 24.**
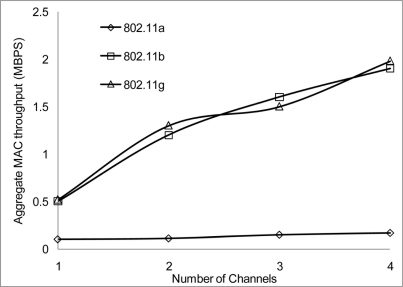
Aggregate throughput at 50 m range and data rate of 2 Mbps.

**Figure 25.**
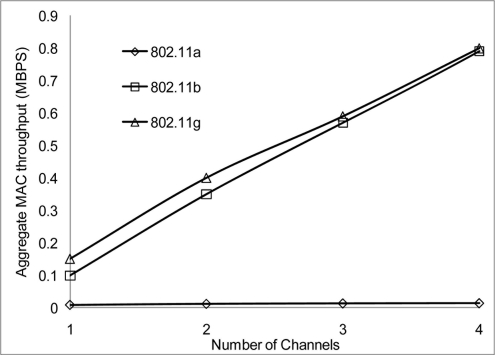
Aggregate throughput at 100 m range and data rate of 2 Mbps.

**Figure 26.**
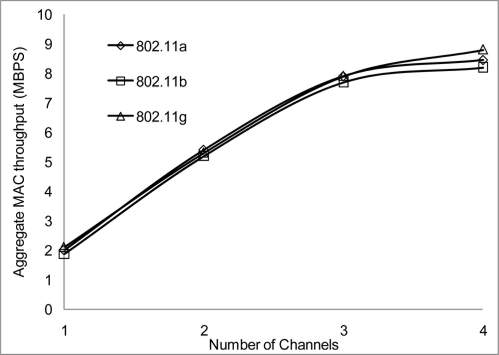
Aggregate throughput at 50 m range and data rate of 10 Mbps.

**Figure 27.**
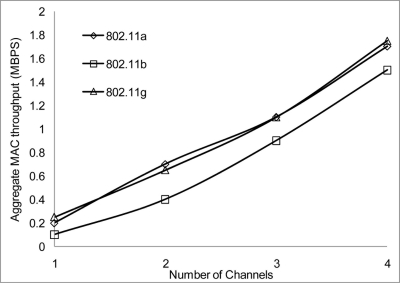
Aggregate throughput at 100 m range and data rate of 10 Mbps.

**Figure 28.**
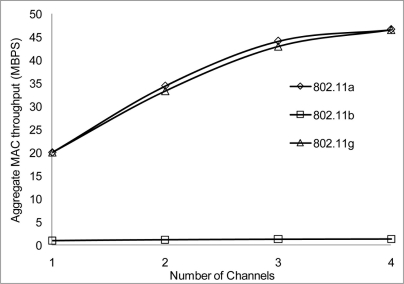
Aggregate throughput at 50 m range and data rate of 54 Mbps.

**Figure 29.**
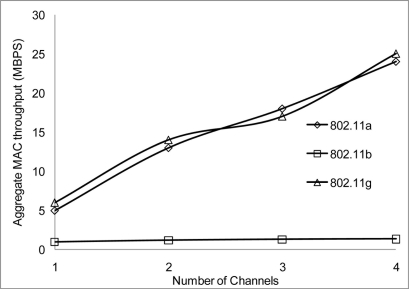
Aggregate throughput at 100 m range and data rate of 54 Mbps.

Figure 24 and 25 show that 802.11 a performed worse with 2 Mbps data rate. Figure 26 show all network performance at 10 Mbps have slight variations with a maximum throughput of 8.8 Mbps when operating over 4 non-overlapping channels. The results showed that with streaming data every 2 seconds with more than 1 channel at data rate of 10 Mbps with the option to switch channel can yield a high performance among all the networks. However, Figure 27 shows significant network degradation when operating at the 100 m range with aggregate throughput within the range of 0.1–1.75 Mbps. Figure 28 and 29 with data rate of 54 Mbps with 802.11 b showed no performance reaction; again this contributes to the maximum data rate of 11 Mbps for 802.11 b.

**Figure 30.**
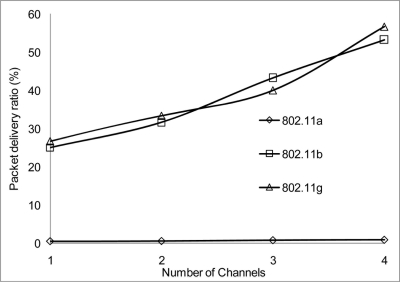
Delivery ratio at 50 m range and data rate of 2 Mbps.

**Figure 31.**
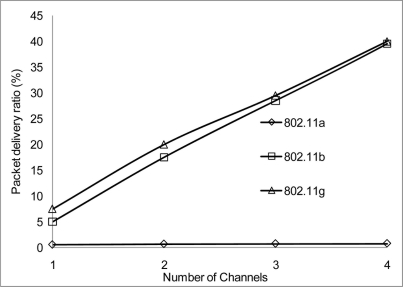
Delivery ratio at 100 m range and data rate of 2 Mbps.

**Figure 32.**
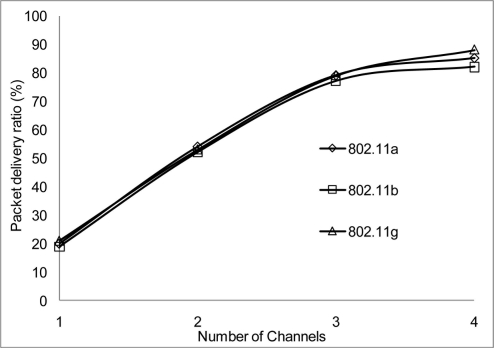
Delivery ratio at 50 m range and data rate of 10 Mbps.

**Figure 33.**
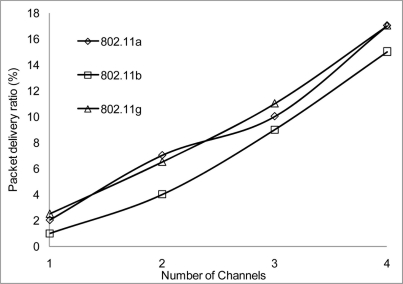
Delivery ratio at 100 m range and data rate of 10 Mbps.

**Figure 34.**
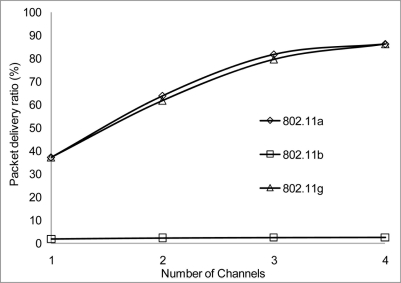
Delivery ratio at 50 m range and data rate of 54 Mbps.

**Figure 35.**
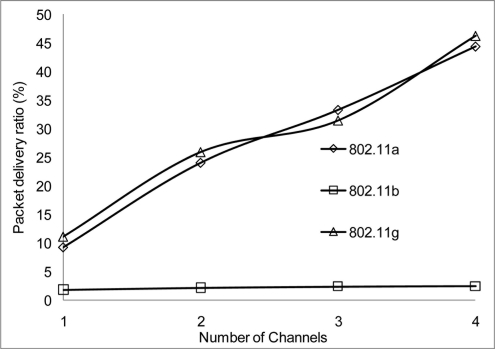
Delivery ratio at 100 m range and data rate of 54 Mbps.

The packet delivery ratio results are shown below in Figures 30–35 as a function of the number of 4 overlapping channels. The results are similar to that of the aggregate throughput, in that, the more channels involve for transmission, the more packets are delivered. More packets are delivered when the range 50 m, at 10 Mbps in Figure 32 all networks delivered almost similar number of packets ranging between 20–87% delivery rates.

Figure 30 shows that under 10% delivery rate for 802.11a as it does not perform well under 6 Mbps. Similarly, 802.11 b in Figure 34 delivery rate was under 10% at 54 Mbps. This is because 802.11 b has a maximum raw data rate of 11 Mbps. All networks perform poorly under 50% delivery rate when operating at 100 m range at 2, 10 and 54 Mbps data rate as can be seen in Figures 31, 33 and 35.

This clearly showed that contention based network perform poorly when the communication range exceeds 50 m. Moreover, the additional overhead experienced during channel switching along with the distance range affect the performance. There are many factors that can influence the date delivery performance in wireless network with no exception to WSNs: the environment, network topology, traffic patterns, *etc.* Also, the 2.4 GHz frequency band is already overcrowded with activities of other networks sharing the same unlicensed band. WSN gives a better performance at short range and with continuous streaming data long range transmission may experience many of the mentioned factors which result in poor performance and as such long range transmission not recommended for WSN.

## 5. Conclusions

In this paper, we proposed MC-DCF that is a backoff algorithm for multi-channel access based on the 802.11 DCF protocols. This algorithm allows node to have access to multiple non-overlapping channels by accessing channels dynamically through channel switching after a set threshold has been met. During the MC-DCF design, we analyzed and discussed the need for multi-channel assignment in WSN, where the future sensor surveillance system with streaming data may find it difficult to operate in 802.15.4 network due to congestion of the most frequently used 2.4 GHz frequency band. The results from the simulation results proved futile for future development in this area for 802.11 networks. We observed that better performance is achieved when using MC-DCF in analyzing the impact of WSN in the 802.11 network. We further tested MC-DCF in 802.11 a/b/g networks at different distance and rates. We observed that at the 50 m range with 10 Mbps all network performed well. Overall 802.11 g performed well with all data rate and this is because it has the additional legacy for backward compatibility with 802.11b, up to 80% delivery rate was obtained.

Overall, MC-DCF exhibited prominent ability to utilize multi-channel transmission for the future with 802.11 for wireless sensor surveillance system that is low-cost, reliable, easy to manage, easy to deploy and can process video data for automated real-time alerts. Researchers will be able to achieve the goal of long term, independent operation of sensor network deployments under this constraint. Also 802.11 will be able to operate within the same frequency band in the capacity of 802.15.4 which is predicted to encounter severe problems when the proposed 802.11n networks become popular.

In the future, we plan to setup testbed sensor network systems and evaluate the MC-DCF performance. We also plan to address the high switching delay experience at the sink node when receiving data from multiple sources.
